# Advanced Practice Providers as Leaders of a Rapid Response Team: A Prospective Cohort Study

**DOI:** 10.3390/healthcare10112122

**Published:** 2022-10-25

**Authors:** Herman G. Kreeftenberg, Ashley J. R. de Bie, Jeroen T. Aarts, Alexander J. G. H. Bindels, Nardo J. M. van der Meer, Peter H. J. van der Voort

**Affiliations:** 1 Department of Intensive Care, Catharina Hospital, Michelangelolaan 2, 5623 EJ Eindhoven, The Netherlands; 2 Executive Board Catharina Hospital, Michelangelolaan 2, 5623 EJ Eindhoven, The Netherlands; 3 TIAS School for Business and Society, Warandelaan 2, 5037 AB Tilburg, The Netherlands; 4 Department of Critical Care Medicine, University Medical Center Groningen, Hanzeplein 1, 9713 GZ Groningen, The Netherlands

**Keywords:** rapid response team, advanced practice provider, physician assistant, intensive care medicine, critical care, outcome, medical resident

## Abstract

In view of the shortage of medical staff, the quality and continuity of care may be improved by employing advanced practice providers (APPs). This study aims to assess the quality of these APPs in critical care. In a large teaching hospital, rapid response team (RRT) interventions led by APPs were assessed by independent observers and intensivists and compared to those led by medical residents MRs. In addition to mortality, the MAELOR tool (assessment of RRT intervention), time from RRT call until arrival at the scene and time until completion of clinical investigations were assessed. Process outcomes were assessed with the crisis management skills checklist, the Ottawa global rating scale and the Mayo high-performance teamwork scale. The intensivists assessed performance with the handoff CEX recipient scale. Mortality, MAELOR tool, time until arrival and clinical investigation in both groups were the same. Process outcomes and performance observer scores were also equal. The CEX recipient scores, however, showed differences between MRs and APPs that increased with experience. Experienced APPs had significantly better situational awareness, better organization, better evaluations and better judgment than MRs with equal experience (*p* < 0.05). This study shows that APPs perform well in leading an RRT and may provide added quality over a resident. RRTs should seriously consider the deployment of APPs instead of junior clinicians.

## 1. Introduction

A combination of increasing demand for healthcare, an accompanying demand for quality and a shortage of medical staff poses a continuous burden for the continuity of healthcare including critical care. In critical care, much of this care is performed by junior clinicians who are regulated by working hours for residents. Moreover, these junior clinicians are not always the best clinicians to counter the problems of complex critical care patients. Advanced practice providers and nurse practitioners are increasingly employed to help meet the demand for quality and continuity in critical care. 

Research in this area supports the supposition that the quality and continuity of care may be sustained by employing advanced practice providers (APPs) [1,2]. Published reports demonstrated that the APPs’ clinical performance was non-inferior to the performance of physicians [3,4]. Besides the non-inferiority, there is increasing evidence on the advantages of employing APPs in critical care. The advantages of these APPs have been assessed by measuring the quality of technical and non-technical skills in the critical care environment and comparing qualities with several other clinicians in the surgical and medical domain [5,6,7,8,9,10,11]. One of the tasks that combines several skills necessary for critical care is participation in a rapid response team (RRT). This team is an in-hospital emergency team that provides critical care for deteriorating patients on wards and arranges transfer to critical care wards if necessary [12]. At the moment, the optimal composition of an RRT is not yet known; the evaluation of rapid response teams emphasizes experience rather than a certain composition, although involvement of intensive care professionals may be beneficial [13,14,15]. A recent study using propensity score matching found that the participation of an APP in an RRT resulted in a decreased length of hospital stay [16]. Moreover, a recent study showed that APPs often provide direct additional value compared to a physician, as it measured process-related outcomes of APPs in a simulated rapid response team environment and showed that an APP might perform better than a medical resident (MR) [17]. 

Although several studies have been performed to assess differences in the level of technical and non-technical skills of APPs, it remains difficult to establish the specific individual contribution of an APP’s performance to the combined outcome of a complex multidisciplinary process of care. Previous studies suggested that this topic should be analyzed in prospective studies using more sophisticated statistical methods [18,19]. 

The aim of this study was to determine whether the process-related performance outcomes of APPs are equal or superior to those of MRs in a rapid response team. We aimed to determine whether we could establish differences between clinicians, in which domains of process performance these differences existed and to what extent the several participants could recognize these outcomes.

## 2. Methods

### 2.1. Design

A single-center prospective observational cohort study was performed in a large teaching hospital (Catharina Hospital in Eindhoven, The Netherlands). In this hospital, all specialties are available, except for neurosurgery and transplant surgery. The study period lasted from 1 April to 30 September 2018. An RRT system has been operational since 2009 as a result of the COMET trial, and such a system is mandatory by law [20]. The RRT is activated by the hospital wards by means of a modified early warning score (EWS) [21]. The efferent limb of the RRT system consists either of an APP or an MR accompanied by an ICU nurse. The clinician carries a pager and can be contacted directly by physicians and nurses in the hospital, based on the EWS. In addition, an intensivist is available for consultation or physical presence if deemed necessary by the RRT.

### 2.2. Population

Posters were displayed at the ICU to encourage all nurses, residents and APPs to contact the independent observers/researchers to enable the observers to assess the RRT visits. The RRT calls could originate from all wards in the hospital including the emergency ward. The resident or APP carrying the pager answered these calls, and when they visited the wards, they called the independent researchers to join them on their visit. Visits were excluded from analysis if they required the physical presence of an intensivist to prevent bias in the independent validation of team members by the researcher.

### 2.3. Power Analysis

No studies were available for power analysis pre-testing. Based on the mean RRT calls per month, we chose to collect data on a minimum of 50 RRT calls in a 4-month period.

### 2.4. Ethical Considerations

The study was approved by the ethics committee (Medical Research Ethics Committees United, Nieuwegein). An informed consent waiver was granted (W17.09) as this study did not change the actual working process or the level of care. Local permission for the study was granted by the executive board of the Catharina Hospital. The procedures followed were in accordance with the ethical standards of the Dutch committee on human experimentation (CCMO) and the above-mentioned ethics committee and with the Helsinki Declaration of 1975.

### 2.5. Data Collection

The observers (independent researchers) were three final-year medical students with clinical experience and knowledge about RRTs. The three students served as independent assessors and tried to attend evening, night and weekend shifts on a voluntary basis within the legal limits. One student registered the results in the database. The three students were trained by two physicians (H.G.K. and A.J.R. deB.) to assess RRT calls. The training consisted of watching three videos of simulated RRT visits led by different APPs and MRs. The evaluation and registration were performed by all three students together to facilitate achieving adequate interobserver results. During the second part of the training, the students evaluated another two simulated RRT visits and assessed them separately. An interobserver variation was obtained to assess the inter-observer variability. The goal was to obtain an interobserver variability of >0.7. In the Ottawa global rating scale, the interclass value proved too low. To address this problem, the students were requested to assess another two videos together and, thereafter, to evaluate two videos separately. The pathology and the teams in the scenarios to assess differed. The results of the interclass correlation for the Mayo high-performance teamwork scale, the Ottawa global rating scale and the crisis management skills were all acceptable to good, with Cronbach alpha values from 0.75 to 0.96.

During the study, the investigators were called by the RRT participants before the RRT visit to join the RRT. The investigators observed the team without intervening the process.

Items registered were time until the assessment of airway, breathing, circulation and neurological status and time until diagnosis. Process outcomes were measured with the Ottawa crisis management skills checklist, the Ottawa global rating scale and the Mayo high-performance teamwork scale (appendix C:1–3) [22,23]. Twenty-four hours after the visit, the MAELOR tool was registered, which served as a validated tool to assess the quality of the RRT visit using the early warning score as reference [21,24]. 

The RRT leader assessed his own performance using the Ottawa global rating scale and the Mayo high-performance teamwork scale. The leaders also assessed their overview over the situation and their handover on the ICU using the handoff CEX “provider” questionnaire (appendix C:4) [25]. This questionnaire is a validated tool to assess the organization, communication, clinical assessment and professionality during the handover of the patients to other clinicians.

The accompanying ICU nurse and the ward nurse who initiated the RTT call were also asked to complete the Ottawa global rating scale and Mayo high-performance teamwork scale

The supervising intensivist completed the handoff CEX “recipient” (appendix C:4) [25]. The specific questionnaires are included in Supplement S1.

All the results were collected in an SPSS^®^ 25 (IBM. Corp., Armonk, NY, USA) and Excel database on a secure hospital server. In addition, the following data were registered: laboratory results, vital parameters, comorbidity, consecutive early warning scores, diagnosis at admission, mortality, data of admission and discharge and DNR code.

These data were used to calculate the Charlson comorbidity index to assess the prognosis, as well as the (quick)SOFA score to assess severity of illness [26]. The patients’ data were collected until discharge from the hospital or for three months.

The MAELOR tool was used to assess the quality of the rapid response team intervention. This tool comprises decisions about “do not resuscitate” and early or late admissions to the ICU of patients with high early warning scores.

### 2.6. Study Outcomes

#### 2.6.1. Primary Outcome

The primary outcome was the difference in the quality of performance of the RRT with either an APP or an MR as the RRT leader. The quality of performance was measured with three patient outcome parameters: MAELOR score, 30-day mortality and hospital mortality.

In addition, process-related outcomes were assessed in 4 ways: (1) the assessor completed the crisis management skills checklist, the Ottawa global rating scale and the Mayo high-performance teamwork scale; (2) the APPs and MRs completed the Ottawa global rating scale and the Mayo high-performance teamwork scale as well as the handoff CEX “provider” questionnaire; (3) the ICU nurse participating in the RTT and the nurse initiating the RTT call completed the Ottawa global rating scale and the Mayo high-performance teamwork scale and (4) the responsible intensivist completed the handoff CEX “recipient” questionnaire. 

#### 2.6.2. Secondary Outcomes

As secondary outcomes, the quality of performance of the RTTs led by APPs versus those led by MRs was measured in three ways: (1) time from RTT call until arrival of the RTT at the patient’s bed; (2) time from RTT call until assessment of airway, breathing, circulation and Glasgow coma score; (3) time from RTT call until diagnosis and diagnostic evaluations.

#### 2.6.3. Statistical Analysis

A two-way, random-consistency intraclass correlation coefficient was calculated with SPSS^®^ 25 (IBM. Corp., Armonk, NY, USA). The use of either parametric or non-parametric distribution was determined with the Kolmogorov–Smirnov test. Dichotomous variables were analyzed with the chi-square test, parametric data with the independent t-test and continuous non-parametric data with the Mann–Whitney U-test. A *p*-value of *p* < 0.05 was considered statistically significant.

## 3. Results

### 3.1. Participants and RRT Calls

A total of 5 APPs and 16 residents participated in the study. The APPs had a median of 5 months experience, and the residents had a median of 6 months experience.

During the study period, a total of 247 RRT calls were made, leading to 247 RRT interventions. Of these interventions, 10 were excluded from analysis because the RRT leader forgot to call the investigator, and 177 were excluded from analysis because no observer was present despite efforts to cover shifts during the day, evening and night. The remaining 60 RTT interventions were assessed by the observers and included in the analysis. Of these interventions, 20 were led by one of the 5 APPs and 40 by one of the 16 residents. 

Of the collected variables, only the qSOFA score had a parametric distribution, and this score is presented as mean with confidence interval. All other data had a non-parametric distribution. There were no significant differences between the participants’ characteristics or between the patients’ characteristics (Table 1). 

### 3.2. Patient-Related Outcomes

The primary outcome showed no significant differences in the quality of RRT interventions between the APP group and the MR group based on the MAELOR tool (*p* = 0.06). In three cases, the APP left the patient in the standard nursing ward with treatment advice, followed by an EWS repeat RRT call after 24 h. One of these patients had yet to be admitted to the ICU. All three patients survived. Mortality was 25% in the APP group versus 17.5% in the MR group, but this difference was not statistically significant (Table 2).

### 3.3. Team-Related Outcomes

There were no statistical differences in time from call until arrival; time from call until assessment of airway, breathing, circulation, neurological evaluation and diagnosis or time from call until admission to the ICU. The results of the CEX provider scale showed that the MRs tended to rate themselves more highly than the APPs did (*p* = 0.037). 

The outcomes of the Mayo high-performance teamwork scale and the Ottawa global rating scale of the observer and the crisis management skills checklist showed no differences either. The outcomes of the CEX provider scale showed that the APPs tend to grade their performance more conservatively than the residents (Table 2). 

**Figure 1:**
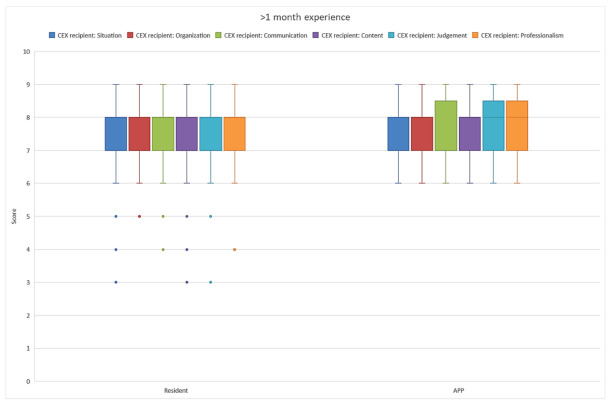
The handoff CEX recipient scores of MRs and APPs with more than 1 month experience.

**Figure 2:**
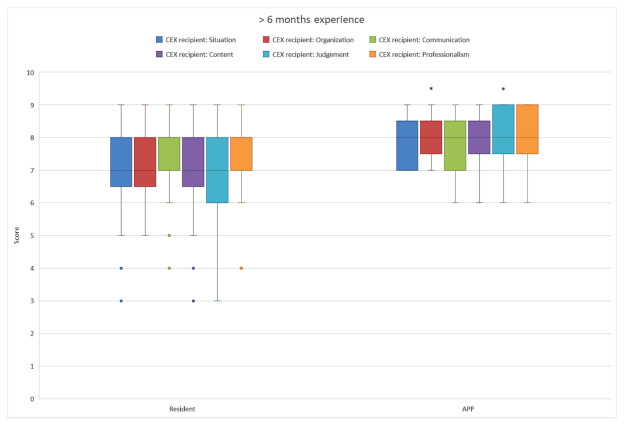
The handoff CEX recipient scores of MRs and APPs with more than 6 months experience. *: *p* < 0.05.

**Figure 3:**
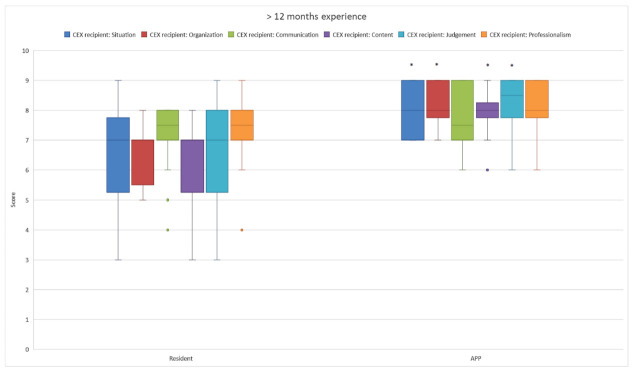
The handoff CEX recipient scores of MRs and APPs with more than 12 months experience. *: *p* < 0.05.

Outcomes of the CEX recipient questionnaire showed that the APPs and MRs initially performed equally well, although the MRs had more outliers to the lower scores (Figure 1). A separate comparison of equally experienced APPs and residents showed that, with increased experience, the APPs performed significantly better in an increasing number of domains (Figure 1, Figure 2 and Figure 3). After 6 months, the CEX recipient scores on organization and judgment of the APPs were significantly better than those of the MRs (Figure 2). When APPs and MRs had more than 1-year ICU experience, the APPs’ overall accumulated CEX recipient questionnaire outcomes also were significantly higher than those of the MRs.

## 4. Discussion

This study found no significant difference in the quality of the RRT visits between teams led by APPs or by MRs regarding patient-related outcomes using mortality and the MAELOR tool. Both types of teams seem to perform equally well, and both teams are non-inferior to each other. The process outcomes such as teamwork communication and general handling of the call, assessed by the crisis management skills checklist, the Mayo high-performance teamwork scale, the Ottawa global rating scale and assessed by the observers and nurses, also showed an equal performance of both groups.

The finding that patient-related outcomes of APPs are non-inferior to those of physicians is in line with the literature [3,4]. However, when we assess the performance of APPs by process outcomes, the handoff CEX recipient scale, an assessment of the RRT procedure in several domains, such as content, organization, judgment and communication as assessed by an experienced intensivist, did show a better performance of the APPs. This better performance of the APP-led teams on the CEX recipient scale is in line with a recent study, which found improved performances in the non-technical skills of APPs compared to physicians [17]. The CEX recipient scale outcome acknowledges the impression that an experienced APP performs on a higher level than an experienced resident. Considering that most critical care delivery is performed by junior physicians, this study suggests that the quality of critical care may be improved by the presence of APPs in rapid response teams.

In addition, the urge of the resident to have a higher esteem of himself than the APP, as shown in the CEX provider scale, raises the question of whether the “humbler” approach of the APP is also a factor in teamwork. Some literature supports this “humble”-approach hypothesis [27]. 

For practical purposes, this study can be used as a model in which an advanced practice provider provides continuity and, therefore, quality of care. Together with a consistent team, these trainable and capable advanced practice providers can enhance the quality of rapid response teams, which often encounter a variety of critical care situations. Their participation enables the supervision of these teams without the physical presence of specialists with a lot of other tasks at hand and guarantees the quality of a rapid response team led by clinicians trained to cope with critical care situations.

### Strengths and Weaknesses

This study is one of the few prospective studies comparing specific skills in APPs versus physicians and the first one for assessing their role as the leader in a rapid response team. Moreover, it also is one of the few studies that measured both patient- and process-related outcomes. The patient-related outcomes of residents and APPs, both supervised by medical specialists, are already inherently good, which makes it difficult to establish differences for these outcomes. The process-related outcomes may, therefore, be better outcomes to measure quality differences between APPs and residents.

The most apparent limitations are inherent to the study design and financial limitations. This was a single-center study because the clinical roles and tasks of APPs in critical care differ between ICUs. They also frequently do not participate in an RRT as a leader. This makes it difficult to have a homogenous intervention group of APPs. In addition, a proper power analysis was not possible since literature guidance was scarce. Therefore, a random time period was chosen to include a minimum number of patients during day, evening and night shifts based on the retrospective evaluation [4]. Unfortunately, only 60 of the 247 RRTs could be observed during the study period, even though a voluntary attempt was taken by the three observers to cover multiple evening, night and weekend shifts. Funding for total coverage was not available. The limited funding combined with the unplannable nature of RRT consultations and the variation of the clinical roles and responsibilities of APPs makes it difficult to perform a multicenter trial about this topic. The RRT consultations that were observed were a random selection and did not introduce bias to the analysis. Therefore, we believe that the results of our single-center study are of value, especially for hospitals that experience difficulties with obtaining enough qualified and experienced physicians to cover all duties of their critical care departments and RRTs. Although the observers were trained to evaluate RRT consultation, they were medical students with limited clinical experience in these acute care scenarios. This limited experience might have influenced their observations in which certain valuable qualities may have remained unnoticed. The better assessment by the experienced intensivist might be a result of these underestimated results. The qualities of the APP have its origin in the focus on critical care: they are often longer acquainted with the critical care environment, and they are familiar with protocol and critical illness assessment in contrast to the medical resident who is often pursuing a career outside critical care. Although tried-and-tested assessment tools were used, other elements, for example emotional control of the clinician and patient perception, were not tested. As these and other process-related outcomes are important and may play a role, this can influence outcome.

Finally, the applied tools to assess the non-technical skills were not specifically developed for RRT consultations but, for example, for simulation studies. This could have influenced the results, since in real life, it is difficult to assess the encountered acute clinical problems using strict rules. 

Nevertheless, this study is in line with other studies about patient and technical skill outcomes. Although APPs often perform relatively more technical and non-technical skills when comparing the number of APPs to the number MRs, this study underscores the difficulty of assessing the APP without their team and using the right tools to measure the added value of an APP [12,28,29,30]. 

Future research could zoom in on the non-technical skills required during these encounters with deteriorating patients and the assessment of these skills by experienced clinicians. Simulation environments maybe practical for these situations because they enable the APPs to boost their non-technical skills in a safe environment. Simulation environments can be adapted to suffice for a local situation, but care must be taken to make these situations as practical and real as possible.

## 5. Conclusions

In this prospective observational study, we confirmed that APPs can provide non-inferior care compared to medical residents during RRT visits. Moreover, the process outcome measures, evaluated by experienced clinicians, show that, even when the standard of care is high, an APP may still improve the quality of care. 

## Figures and Tables

**Table 1:** Baseline characteristics of patients treated by either an APP or an MR.

Characteristics	APP	MR
Experience of the clinician in months *	5 (1–104)	6 (2–17)
Reason for RRT call		
Respiratory or airway problems	12	11
Hemodynamic instability/sepsis **	7	21
Electrolyte disorders	1	1
Altered consciousness	0	1
Other	0	6
Specialism		
Surgery	7	6
Internal medicine	3	13
Cardiology	3	3
Pulmonology	2	2
Cardiothoracic surgery	2	1
Gastroenterology	0	3
Neurology	3	1
Other	0	11
Admitted to the ICU	13	25
Patient qSOFA	0.95	1.2
Patient age *	69 (63–80)	68 (55–78)
Patient gender male	20	26
Patient gender female	8	14
Age-adjusted Charlson comorbidity index *	7 (3–8.8)	4.5 (3–6.8)

*: Median and interquartile range. **: The separate diagnoses did not differ significantly between the groups.

**Table 2:** Patient- and process-related outcomes of patients treated by either an APP or an MR.

Patient-Related Outcome	APP	MR	*p*-Value (Chi Square)
Mortality (30 day)	5/20 (25%)	7/40 (17.5%)	0.73 (0.117)
MAELOR positive	17	40	0.59 (3.55)
Admission < 4 h	13	25	na
No admission	6	15	na
Admission > 4 h	1	0	na
Process-Related Outcome *	APP Median (IQR)	MR Median (IQR)	*p*-Value (u-Value and z-Score)
Time between call and arrival (min:sec)	4:30 (2:45–9:30)	3:59 (2:00–6:00)	0.28 (258.5, −1.07)
Time until respiratory exam (min:sec)	0:30 (0:00–1:00)	0:00 (0:00–1:00)	0.25 (318.5, −1.14)
Time until circulation exam (min:sec)	2:00 (1:00–4:00)	1:00 (0:44–3:00)	0.13 (289, −1.53)
Time until EMV exam (min:sec)	2:00 (1:00–4:00)	1:00 (0:00–3:00)	0.91 (336, −0.11)
Time until diagnosis (min:sec)	5:00 (3:00–10:00)	5:00 (3:00–8:00)	0.72 (276.5, −0.36)
Crisis management skills total	27 (25–28)	27 (26–28)	0.59 (366.5, −0.53)
MAYO observer	22 (22–24)	23 (22–24)	0.34 (340.5, −0.95)
GRS observer	6 (6–7)	6(6–7)	0.96 (397.5, −0.048)
MAYO ICU nurse	21 (19–24.5)	24 (20.7–25)	0.22 (57.5, −1.22)
GRS ICU nurse	6 (6–6.5)	6 (6–6)	0.44 (69.5, −0.77)
CEX provider	7 (6–7.25)	7 (7–8)	0.04 (233, −2.09)

*: All time data as minutes:seconds.

## Supplementary Materials

Supplement S1. assessment scales.

## Abbreviations

APP	Advanced practice provider
EWS	Early warning score
ICU	Intensive care unit
MR	Medical resident
RRT	Rapid response team
